# Perceiving emotion in non-social targets: The effect of trait empathy on emotional contagion through art

**DOI:** 10.1007/s11031-017-9619-5

**Published:** 2017-05-18

**Authors:** Olga Stavrova, Andrea Meckel

**Affiliations:** 1 Department of Social Psychology, Tilburg University, P.O. Box 90153, 5000 LE Tilburg, The Netherlands; 2 Leibniz-Institute for the Social Sciences, Mannheim, Germany

**Keywords:** Empathy, Emotional contagion, Music, Poetry, Art

## Abstract

This research examines the role of trait empathy in emotional contagion through non-social targets—art objects. Studies 1a and 1b showed that high- (compared to low-) empathy individuals are more likely to infer an artist’s emotions based on the emotional valence of the artwork and, as a result, are more likely to experience the respective emotions themselves. Studies 2a and 2b experimentally manipulated artists’ emotions via revealing details about their personal life. Study 3 experimentally induced positive vs. negative emotions in individuals who then wrote literary texts. These texts were shown to another sample of participants. High- (compared to low-) empathy participants were more like to accurately identify and take on the emotions ostensibly (Studies 2a and 2b) or actually (Study 3) experienced by the “artists”. High-empathy individuals’ enhanced sensitivity to others’ emotions is not restricted to social targets, such as faces, but extends to products of the human mind, such as objects of art.

## Introduction

In today’s psychological literature, empathy is understood as a predominantly social phenomenon, describing individuals’ sensitivity to others’ emotions and mental states (Eisenberg and Fabes 1990; Zaki 2014). As an important component of social cognition, it is believed to facilitate communication, encourage prosocial behavior and ensure social cohesion (Balconi and Canavesio 2013; Batson et al. 1991; Batson and Moran 1999; Lim and DeSteno 2016; Oceja et al. 2010; Prot et al. 2014; Winczewski et al. 2016). However, historically, empathy has been considered mainly in non-social contexts. The term ‘empathy’ (derived from the German word ‘*Einfühlung*’ meaning ‘*feeling into*’) was coined by Theodor Lipps (1903) and translated into English by Edward Titchener (1909), to explain people’s aesthetic appreciation of art. As the opening quotation illustrates, in this early view, empathy described the tendency to empathize with objects, see objects as part of the self (Freedberg and Gallese 2007; Gladstein 1984). Despite this early theorizing, most existing empirical research on empathy has focused on its role in social contexts (Decety and Cowell 2014; Engelen and Röttger-Rössler 2012; Zaki 2014), whereas the effect of empathy on individuals’ perception of non-social targets, including art, remained substantially less explored.

Herein, we examine individual differences in trait empathy in emotional reactions to art, using the examples of music, photography and poetry. Given high-empathy individuals’ enhanced attention and sensitivity to others’ emotional states (Engelen and Röttger-Rössler 2012; Zaki 2014), we propose that while contemplating artworks, high-empathy individuals are more likely to experience the emotions expressed in the artwork than their low-empathy counterparts. We also investigate the mechanism behind this effect. We suggest that individuals high in trait empathy are more likely to use emotional cues contained in the artwork to make inferences about the artist’s emotions, which results in their stronger emotional sensitivity to art.

## Empathy and emotional contagion

Empathy is a broad concept comprising multiple phenomena, mostly pertaining to individuals’ ability to accurately perceive and take on others’ mental states (Preston and Hofelich 2012). Individuals high in trait empathy have been shown to be more likely to correctly identify other people’s mental states from their eyes (Baron-Cohen et al. 2001) and to accurately identify others’ emotions based on their facial expressions (Dimberg et al. 2011; Gery et al. 2009; Svetieva and Frank 2015; Zaki et al. 2008). Another important aspect of empathy is the tendency to take on others’ motor, visceral and affective states, e.g., to experience emotions experienced by others (Decety and Cowell 2014). The process of contracting others’ emotions is also referred to as *emotional contagion* (Hatfield et al. 1993) or *cross-over effects* (Westman 2013). Multiple studies have shown individual differences in empathy to be associated with susceptibility to others’ emotions (Dimberg et al. 2011; Wiesenfeld et al. 1984). For example, in organizational settings, high-empathy individuals were shown to be more likely to be subject to emotional contagion of positive emotions from their team leaders than low-empathy individuals (Westman et al. 2013). The proneness of empathetic individuals to emotional contagion has been evidenced with physiological and brain imaging data as well. For example, high-empathy individuals are more likely to mimic emotional expressions displayed by target faces than low-empathy individuals, as indicated by increased facial electromyographic activity in the respective muscles (Dimberg et al. 2011; Sonnby-Borgström 2002). When exposed to their loved one in pain, high-empathy individuals are more likely to show an activation in brain areas involved in the processing of pain than low-empathy individuals (Singer et al. 2004).

Research linking trait empathy with emotional contagion has mainly considered contagion via in-person interactions, e.g., when recipients are exposed to targets’ emotions either in a direct interaction or via photographs/videos. Interestingly, most recent research on emotional contagion has shown that emotions experienced by one person can spread to others without any direct contact between them (Fowler and Christakis 2009; Kramer et al. 2014; Rosenquist et al. 2011; Stavrova 2015). For example, analyses of social networks data showed that an individual’s emotions do not only “contaminate” the emotions of this individual’s friends’ but also the emotions of his/her friends’ friends (Fowler and Christakis 2009).

If an individual can “catch” the emotions of an unrelated person via exposure to her tweets or social networks status updates, art objects might represent another medium through which emotions can be transferred from one person (the artist) to another (the observer). The psychological literature on aesthetics and art has emphasized the importance of empathy and perspective taking—speculating about the artist’s mind, intentions and plans—for art appreciation (Bloom 2004; Hawley-Dolan and Winner 2011; Newman and Bloom 2012). Both experimentally induced and dispositional empathy have been shown to facilitate the perception of artists’ expressive intentions in music and dance (Sevdalis and Keller 2011; Wöllner 2012). For example, empathy in jazz musicians was positively associated with their accuracy of categorizing a recoded melody as an improvisation or an imitation (Engel and Keller 2011). The role of empathy has been highlighted in emotional reactions to music as well (Juslin et al. 2014; Juslin and Västfjäll 2008; Miu and Balteş 2012). High-empathy individuals were shown to be more likely to experience the emotions expressed in music than low-empathy individuals (Egermann and McAdams 2013; Vuoskoski and Eerola 2011, 2012).

The present studies extend this research in a number of ways. First, we explored whether the effect of trait empathy on emotional reactions to art is restricted to music or can be observed with respect to other kinds of art, such as visual art and literary texts. Second, we examined the mechanism of this effect by investigating high- (vs. low-) empathy individuals’ inferences about artists’ emotions. Given high-empathy individuals’ enhanced attention to emotional information (Hofelich and Preston 2012) and ability to understand others’ emotional states (Baron-Cohen et al. 2001; Dimberg et al. 2011), we propose that high-empathy individuals are more sensitive to the emotional cues contained in artworks and are more likely to use them to make inferences about the emotions experienced by the artist, compared to their low-empathy counterparts. As a consequence, high-empathy individuals are more likely to become subject to emotional contagion through art, *i.e*., to experience the emotions expressed in artworks.

Finally, we extended prior research by exploring the role of empathy not only in people’s ability to detect and experience the emotions *expressed* in the artwork, but also the emotions *actually experienced by the artist* during the creation process. While contemplating an artwork, can individuals identify the artist’s emotions at a better than chance level? Do high levels of empathy facilitate this task? The present research was designed to answer these questions.

## The present research

We report the results of five experiments that investigated the role of trait empathy in emotional contagion through art and its mechanism across the domains of music, photography and poetry. We do not restrict our study to professional artists, neither to artifacts “officially” recognized as art. In fact, as the history of modern art (e.g., a urinal by Marcel Duchamp or vacuum cleaners by Jeff Koons) and recent psychological research (Preissler and Bloom 2008) have shown, people can perceive virtually any object as a piece of art when they believe that this is what its author intended it to be. In Studies 1a and 1b, we manipulated the emotions expressed in music (Study 1a) and photographs (Study 1b) and examined whether individual differences in empathy are associated with a stronger tendency to infer an artist’s emotions based on the emotional valence of his or her work. We then explored whether these inferences underlie emotional contagion through art, that is, mediate the effect of the emotional valence of an artwork on individuals’ experienced emotions. To rule out alternative causal explanations, in Studies 2a and 2b, we examined this process using an experimental causal-chain mediation approach (Spencer et al. 2005) by including an experimental manipulation of the mediator, i.e., an artist’s emotions. Finally, in Study 3, we manipulated individuals’ emotional state just before they started working on a literary text. We then showed the obtained texts to another sample of participants and examined whether individual differences in empathy facilitate the identification and experience of the emotions actually experienced by the “artists”.

## Study 1a

In this study, we examined whether individual differences in trait empathy are associated with the tendency to make inferences about a composer’s emotions based on the emotional valence of his or her music and whether these inferences result in high-empathy individuals being more likely to experience the respective emotions themselves, compared to low-empathy individuals. Therefore, we manipulated the emotional valence of the music and measured individuals’ perception of the composer’s emotions, their own experienced emotions and trait empathy.

### Method

For this study, we recruited 120 individuals from Amazon Mechanical Turk (MTurk) (Buhrmester et al. 2011). Ten individuals failed a data quality check question (which required them to select a particular response option instead of answering the question) or indicated that they had technical problems hearing the music and were excluded from the analysis, resulting in a final sample of 110 individuals (mean age 36.47, *SD* = 11.43, 52.7% men). Participants listened to three either happy or sad pieces of music that were selected from a database of music excerpts assembled by Eerola and Vuoskoski (2011b). All stimuli have been pretested to insure that they represent highly typical examples of happiness and sadness (Eerola and Vuoskoski 2011a). For this study, we selected three pieces (app. 60 s each) that were rated as highly happy and three pieces that were rated as highly sad in pretests (excerpts IDs: H1, H3, H4, S1, S3, S4). After each piece, using a scale ranging from 1 = ‘not at all’ to 7 = ‘a lot’, participants stated their current emotions [‘happy’ and ‘sad (reverse-coded)’, Cronbach’s α = 0.87] and, using the same scale, indicated how they thought the composer was feeling while composing the respective piece of music (Cronbach’s α = 0.92). The order in which participants stated their own emotions vs. estimated the composer’s emotions was counterbalanced. As it had no effect on either of the variables and did not interact with the experimental condition (all *p*s > 0.33) we did not consider it in further analyses. After a number of filler items and standard socio-demographic questions,^1^ participants completed the Toronto Empathy Questionnaire (TEQ) (Spreng et al. 2009). As we were interested in both emotional contagion and empathetic accuracy, we selected TEQ as this scale was designed to measure both cognitive and affective facets of empathy. The scale consists of 16 items (eight positively and eight negatively worded; sample items: “When someone else is feeling excited, I tend to get excited too”, “I can tell when others are sad even when they do not say anything”). It has been shown to correlate most strongly with measures of affective empathy (the ability to take on others’ emotions), but to be also related to its cognitive component (the ability to understand what emotions are experienced by the target). The scale has a robust single-factor structure, high internal validity (in this study, Cronbach’s *α* = 0.89) and shows strong correlations with behavioral measures of emotional accuracy (Spreng et al. 2009).

### Results

Means, standard deviations and zero-order correlations are shown in Table 1. To examine whether the effect of the emotional valence of the music on participants’ experienced emotions is moderated by individual differences in trait empathy, we conducted a 2 (music valence: positive vs. negative) x empathy (scale predictor, mean centered) MANOVA with participants’ experienced emotions and perceived emotions of the composer as dependent variables.^2^ The model specified the effects of the emotional valence of the music, participants’ empathy and their interaction. The omnibus test revealed a significant main effect of music valence, Pillai’s Trace *F* (2, 105) = 178.43, *p* < .001, η^2^_partial_ = 0.77, and a significant interaction between music valence and empathy, Pillai’s Trace *F* (2, 105) = 6.29, *p* = .003, η^2^_partial_ = 0.11.

**Table 1:** Means, standard deviations and zero-order correlations among the variables, Studies 1a–2b

		Study 1a/Study 1b					Study 2a / Study 2b					
		*M*	*SD*	1	2	3	*M*	*SD*	1	2	3	4
1	TEQ	2.89/2.93	0.42/0.39	–	–	–	2.94/3.14	0.34/0.49	–	–	–	–
2	Experienced emotions	5.44/4.95	1.29/1.42	0.05/0.02	–	–	4.96/4.23	1.44/1.59	0.02/-0.05	–	–	–
3	Perceived emotions	4.73/4.65	1.8/1.63	0.02/-0.06	0.75***/0.77***	–	4.73/4.65	1.8/1.63	–	–	–	–
4	Artwork’s valence (manip. factor)	0.49/0.50	0.50/0.50	0.40	0.65***/0.61***	0.87***/0.86***	0.51/0.46	0.50/0.50	0.04/-0.03	0.43***/0.75***	–	–
5	Artist’s emotions (manip. factor)	–	–	–	–	–	0.51/0.51	0.50/0.50	−0.13/0.07	0.30***/0.25***	–	0.01

*** *p* < .001. TEQ: Toronto Empathy Questionnaire

#### Perceived emotions

**Fig. 1:**
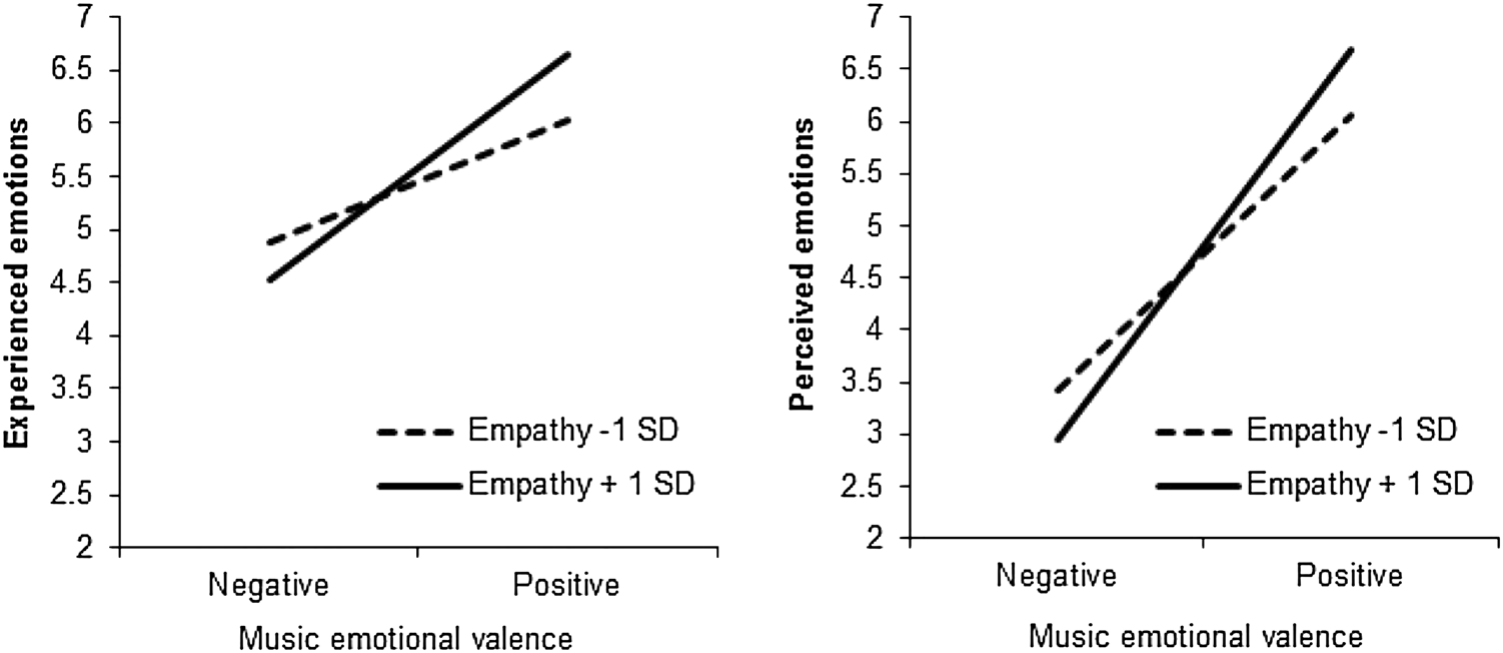
Participants’ experienced and perceived emotions (1 = negative, 7 = positive) as a function of their empathy level and the emotional valence of the music, Study 1a

Participants believed that the composer experienced more positive emotions in the happy music (*M* = 6.36, *SD* = 0.61) than in the sad music condition (*M* = 3.19, *SD* = 1.30), *F* (1, 106) = 359.97, *p* < .001, η^2^_partial_ = 0.77. This effect was stronger for high- (1 *SD* above the mean, *M*_dif_ = 3.73, *p* < .001) than for low- (1 *SD* below the mean, *M*_dif_ = 2.62, *p* < .001) empathy individuals, *F* (1, 106) = 10.84, *p* = .001, η^2^_partial_ = 0.09 (Fig. 1). That is, high-empathy individuals were more likely to infer the composer’s emotions based on the emotional valence of the music than low-empathy individuals.

#### Experienced emotions

With respect to their own experienced emotions, participants reported more positive emotions after listening to happy excerpts (*M* = 6.34, *SD* = 0.60) than sad excerpts (*M* = 4.71, *SD* = 1.23), *F* (1, 106) = 81.09, *p* < .001, η^2^_partial_ = 0.43. This effect was qualified by a significant interaction with empathy, *F* (1, 106) = 7.29, *p* = .008, η^2^_partial_ = 0.06. As Fig. 1 shows, the effect of the emotional valence of the music on participants’ experienced emotions was stronger in participants with a relatively high (+1 *SD*) empathy score (*M*_dif_ = 2.13, *p* < .001) than in participants with a relatively low (−1 *SD*) empathy score (*M*_dif_ = 1.15, *p* < .001). In other words, high-empathy individuals were more likely to become subject to emotional contagion through music, that is, to experience the emotions expressed in the music they listened to.

#### Moderated mediation analysis

**Fig. 2:**
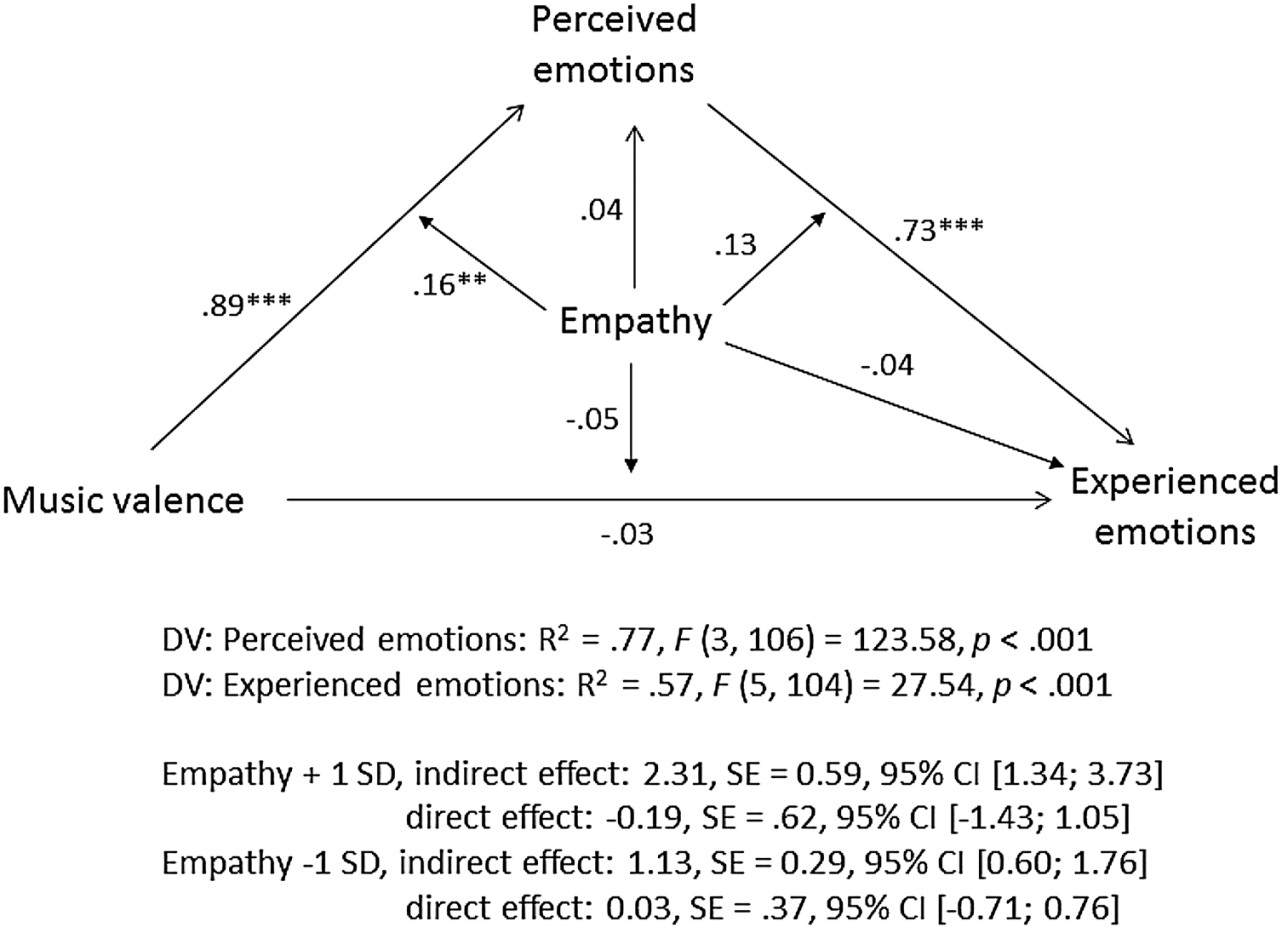
Moderated mediation analysis, Study 1a. Note. The diagram paths present the standardized regression coefficients, the estimation of the indirect effects is based on unstandardized coefficients (Hayes 2016)

To examine whether participants’ perception of the emotions experienced by the composer mediated the emotional contagion effect (i.e., the effect of the emotional valence of the music on participants’ experienced emotions) and whether this mediation was stronger in high- versus low-empathy individuals, we conducted a moderated mediation analysis with music valence as independent variable, experienced emotions as dependent variable, perceived emotions as a mediator and individuals’ trait empathy as a moderator (see Fig. 2). We used model 59 of the process macro by Hayes (2013). All effects are tested simultaneously, including all three interactions (s. Fig. 2). The moderated mediation takes place, if either path “a” (the effect of IV on the mediator), path “b” (the effect of the mediator on the DV) or both are significantly moderated by the moderator variable (that is, by empathy) (Preacher et al. 2007). The effect of the moderator on path c’ (that is, the effect of IV on DV) does not need to be significant in the overall model (Preacher et al. 2007). Confidence intervals were constructed using the bootstrapping method with 5000 re-samples. The model coefficients are presented on Fig. 2. As indicated by a significant music valence by empathy interaction (*b* = 0.51, *p* < .01), high-empathy individuals were more likely to use the valence of the music to make inferences about the composer’s emotions than low-empathy individuals and, consequently, were more likely to experience these emotions themselves, providing first support for the moderated mediation. Indeed, for low-empathy individuals (−1 *SD*), the conditional indirect effect of music valence on experienced emotions through perceived emotions was *b* = 1.10, *SE* = 0.29, 95% CI (.54; 1.71); whereas for high-empathy individuals (+1 *SD*), it was twice the magnitude, *b* = 2.36, *SE* = 0.59, 95% CI (1.40; 3.71). That is, the mediation via the perception of the composer’s emotions was nearly twice as strong among high- compared to low-empathy individuals.

To summarize, high-empathy individuals are more likely to infer a composer’s emotions on the basis of the emotional valence of his or her music than low-empathy individuals and these inferences make them subjects to a stronger emotional contagion effect.

## Study 1b

This study replicated the results of Study 1a with respect to visual art: photography. Like in Study 1a, we examined whether high-empathy individuals are more likely to experience the emotions expressed in photographs and whether this effect is mediated by their inferences about the photographers’ emotions.

Prior research has shown that individual differences in trait empathy are strongly related to the basic dimensions of personality, in particular agreeableness (Graziano et al. 2007; Mooradian et al. 2011), with highly agreeable individuals being more likely to score highly on empathy. In addition, agreeableness (as well as other dimensions of the Big Five, such as extraversion and openness) has been connected to emotional responses to art as well (Vuoskoski and Eerola 2011). Therefore, to make sure that the effect of empathy is not due to a confounding with the Big Five dimensions of personality, in Study 1b we additionally controlled for individual differences in the Big Five.

### Method

Two hundred and seven individuals, recruited on MTurk, participated in this study. Nine failed a data attention check question (which required them to correctly identify the theme of the last photograph they viewed), resulting in a final sample of 198 individuals (mean age 36.1, *SD* = 12.97, 60.6% men). Participants were shown five either positive or negative valence photographs selected from the International Affective Picture System (Lang et al. 2008). We selected the stimuli that were shown to elicit positive versus negative emotions in pretests (specifically, obtained above vs. below scale midpoint scores in valence; stimuli #: 5660, 6000, 5760, 9080, 5811, 5961, 8170, 9001, 5780, 9010) (Lang et al. 2008). Pictures in the negative valence condition (*M* = 4.58, *SD* = 0.82) did not differ from the pictures in the positive valence (*M* = 4.32, *SD* = 1.28) condition on the dimension of arousal, *t* (8) = 0.38, *p* = 0.71. Also, arousal and valence among the selected pictures did not show a substantial correlation (*r* = −0.12, *p* = 0.74). As we were interested in the role of empathy in emotional reactions to non-social targets, we selected photographs presenting nature in different states or urban sceneries and strictly avoided photographs that displayed people or animals (e.g., photographs showing mountains or flower fields were used in the positive emotional valence condition and photographs showing electric wires or tornados were used in the negative emotional valence condition). We used the same measure of participants’ experienced emotions and the same measure of their perception of the artist’s emotions as in Study 1a (Cronbach’s α = 0.93 and 0.95, respectively). Like in Study 1a, the order in which participants completed these measures was counterbalanced and trait empathy was measured with the TEQ (Cronbach’s α = 0.89). As a measure of the Big Five, we used the Mini-IPIP that measures the Big Five dimensions with four items each (Cronbach’s α between 0.74 and 0.84) (Donnellan et al. 2006).

### Results

Means, standard deviations and zero-order correlations are shown in Table 1. We conducted a 2 (photographs’ valence: positive vs. negative) x 2 (item order: experienced emotions measured first vs. perceived emotions measured first) x empathy (scale predictor, mean centered) MANOVA with experienced emotions and perceived emotions as dependent variables. The model included all main effects, three two-way interactions and one three-way interaction between the variables. The omnibus test revealed significant main effects of photographs’ valence, Pillai’s Trace *F* (2, 188) = 290.79, *p* < .001, η^2^_partial_ = 0.76, item order, Pillai’s Trace *F* (2, 188) = 9.11, *p* < .001, η^2^_partial_ = 0.09, a significant interaction between photographs’ valence and item order, Pillai’s Trace *F* (1, 188) = 6.32, *p* < .01, η^2^_partial_ = 0.06, and a significant interaction between photograph’s valence and empathy, Pillai’s Trace *F* (2, 188) = 7.89, *p* < .01, η^2^_partial_ = 0.08. No other effects or interactions reached significance (all *p*s > 0.32).

#### Perceived emotions

**Fig. 3:**
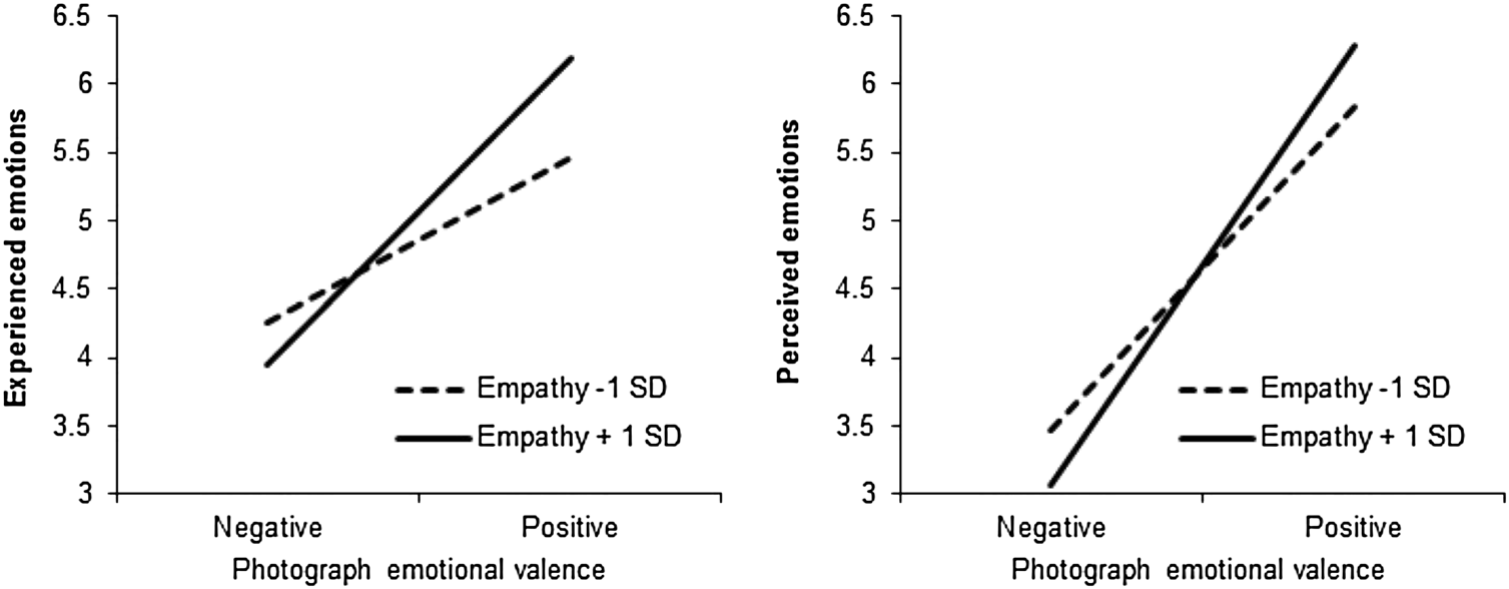
Participants’ experienced and perceived emotions (1 = negative, 7 = positive) as a function of their empathy level and the emotional valence of the photographs, Study 1b

Participants believed the photographer to experience more positive emotions after viewing positive valence (*M* = 6.05, *SD* = 0.74) than negative valence photographs (*M* = 3.27, *SD* = 0.93), *F* (1, 189) = 569.23, *p* < .001, η^2^_partial_ = 0.75. This effect was stronger for high- (*M*_dif_ = 3.23, *p* < .001) than for low- (*M*_dif_ = 2.36, *p* < .001), *F* (1, 189) = 13.45, *p* < .001, η^2^_partial_ = 0.07, empathy individuals (Fig. 3).

#### Experienced emotions

There was a significant main effect of photographs’ valence, *F* (1, 189) = 132.96, *p* < .001, η^2^_partial_ = 0.41. Participants who viewed positive valence photographs reported more positive emotions (*M* = 5.81, *SD* = 0.93) than participants who viewed negative valence (*M* = 4.09, *SD* = 1.30) photographs. This effect was qualified by a significant interaction with empathy, *F* (1, 189) = 12.10, *p* = .001, η^2^_partial_ = 0.06. This interaction is presented on Fig. 3. It shows that the effect of photographs’ emotional valence on participants’ experienced emotions was stronger in participants with a relatively high (+1 *SD*) empathy score (*M*_dif_ = 2.26, *p* < .001) than in participants with a relatively low (−1 *SD*) empathy score (*M*_dif_ = 1.21, *p* < .001).

The interaction between photographs’ valence and item order, significant in the omnibus test (s. above), was restricted to participants’ experienced emotions, *F* (1, 189) = 10.73, *p* < .01, η^2^_partial_ = 0.05; it did not affect perceived emotions, *p* = .37. The effect of photograph’s valence on experienced emotions was weaker if participants’ stated their emotions after rating the photographer’s emotions. Probably, the effect of the photographs’ emotional valence was just more likely to fade due to a longer time span between the exposure to the photographs and measures of experienced emotions in this case. Importantly, as indicated by a non-significant three-way interaction (*p* = .49), all the effects pertaining to empathy were unaffected by this item order effect.

#### Big Five

Next, we examined whether the effect of empathy on participants’ reactions to emotions expressed in photographs and their judgment of photographer’ emotions could have been driven by a confounding with the Big Five. Therefore, we repeated the analyses described above with participants’ Big Five scores as covariates. The results showed that the interaction between empathy and photographs’ valence remained significant when controlling for individual differences in the Big Five, Pillai’s Trace *F* (2, 181) = 8.82, *p* < .001, η^2^_partial_ = 0.09 (perceived emotions:*F* (1, 182) = 13.44, *p* < .001, η^2^_partial_ = 0.07; experienced emotions:*F* (1, 182) = 14.24, *p* < .001, η^2^_partial_ = 0.07).

#### Moderated mediation analysis

**Fig. 4:**
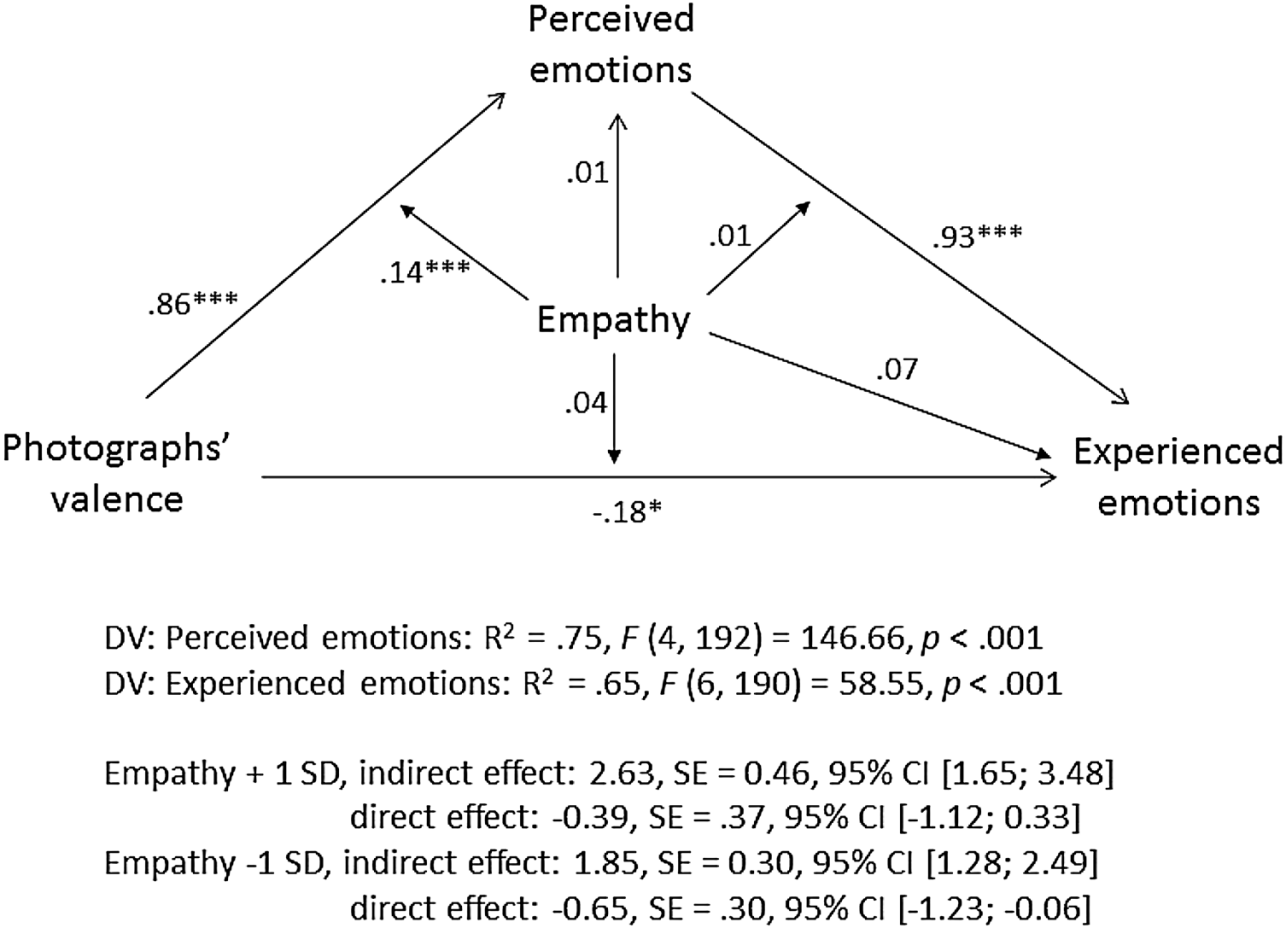
Moderated mediation analysis, Study 1b. Note. The diagram paths present the standardized regression coefficients, the estimation of the indirect effects is based on unstandardized coefficients (Hayes 2016)

Like in Study 1a, to examine whether participants’ inferences about the photographer’s emotions mediated the effect of photographs’ emotional valence on participants’ experienced emotions and whether this indirect effect was stronger in high- vs. low-empathy individuals, we conducted a moderated mediation analysis with photographs’ valence as independent variable, experienced emotions as dependent variable, perceived emotions as a mediator, empathy as a moderator and item order as a covariate (we included item order as it was related to the dependent variable and the mediator, as the analysis of variance described above has shown). We used the same procedure as in Study 1a and the path coefficients are presented on Fig. 4. As indicated by a significant interaction between photographs’ emotional valence and empathy (*b* = 1.08, *p* < .001), high-empathy individuals were more likely to use the emotions expressed in photographs to make inferences about the photographer’s experienced emotions than low-empathy individuals and, consequently, were more likely to experience these emotions themselves. As the look at the indirect effects shows (Fig. 4), for low-empathy individuals (−1 *SD*), the conditional indirect effect of photographs’ valence on experienced emotions via perceived emotions reached *b* = 1.87, *SE* = 0.32, 95% CI (1.26; 2.54); whereas for high-empathy individuals (+1 *SD*), it was substantially larger, *b* = 2.66, *SE* = 0.45, 95% CI (1.78; 3.53). That is, high-empathy (compared to low-empathy) individuals were more likely to infer the photographer’s emotions from the emotions expressed in the photographs and these inferences mediated the effect of expressed emotions on experienced emotions.

## Discussion

Taken together, Studies 1a and 1b have shown that trait empathy is associated with an enhanced ability to perceive emotions in non-social targets—artworks. When exposed to sad (vs. happy) music or photographs, individuals with a relatively high empathy level were more likely to report experiencing the respective emotions themselves than their less empathetic counterparts. Furthermore, high- (compared to low-) empathy individuals were more likely to infer the artist’s emotions based on the emotions expressed in the artwork, be it a piece of music or a photograph. Using a moderated mediation analysis, we have shown that these inferences explained high-empathy individuals’ enhanced emotional reactions to artworks.

These studies used a measurement-of-mediation approach: while allowing the estimation of the indirect effect, this approach has one important limitation (Spencer et al. 2005). Involving a manipulation of the independent variable (artworks’ emotional valence), but not of the mediator (artists’ emotions), it opens a possibility of alternative causal explanations. For example, the emotional valence of the artwork could have affected high-empathy individuals’ experienced emotions in the first place. As prior research has shown individuals’ emotions to guide their judgment (Clore and Huntsinger 2007; Schwarz and Clore 1983), participants’ experienced emotions could have then affected their judgment of the artist’s emotions, rather than another way around. To rule out this alternative explanation, in Studies 2a and 2b, we attempted to establish a causal effect of perceived emotions on participants’ experienced emotions. We employed an experimental-causal-chain mediation design (Spencer et al. 2005) by experimentally manipulating the mediator – artists’ emotions. As our major research question concerns the role of trait empathy, our analysis focused on whether the causal effect of artists’ emotions on participants’ experienced emotions depends on their empathy level.

## Study 2a and 2b

In Studies 2a (music) and 2b (photographs), we manipulated the emotions of the artist (positive vs. negative) and expected this manipulation to result in a stronger effect on participants’ experienced emotions among high- (rather than low-) empathy individuals. A significant interaction between the artist’s emotions and participants’ empathy level will provide evidence for this hypothesis.

Also, manipulating the artist’s emotions might affect individuals’ perception of the emotional valence of the artwork (e.g., individuals might believe that a neutral piece of music expresses happiness or sadness, depending on the composer’s mood). To take care of this potential confounding, we additionally manipulated the emotional valence of the artwork (positive vs. negative) and explored whether the effect of the artist’s emotions on participants’ experienced emotions and, most importantly, its interaction with empathy are independent from the emotions expressed in the artwork.

## Study 2a

### Method

Two hundred and eighteen individuals recruited on MTurk participated in this study. Four participants were excluded as they reported technical problems hearing the music, resulting in a final sample of 214 individuals (mean age 35.57, *SD* = 12.0, 54.4% males). Participants were randomly assigned to one out of four experimental conditions in a 2 (music emotional valence: positive vs. negative) x 2 (composer’s emotions: positive vs. negative) between-subject design. Participants read about a composer Lucas M., who had either just married and celebrated the birth of his first child (composer’s emotions condition: positive) or lost his wife and his new born child in a car accident (composer’s emotions condition: negative). Participants then listened to a piece of music (either sad or happy) ostensibly composed by Lucas M. and dedicated to his family. We used the same pieces of music (we selected one for the positive and one for the negative valence condition) as in Study 1a. After listening to the music, participants indicated to what extent they felt happy, sad, cheerful and upset (on a scale ranging from 1 = ‘not at all’ to 7 = ‘a lot’). The responses were recoded such that higher values reflect more positive emotions and combined into one scale (Cronbach’s α = 0.84). As a manipulation check, participants rated the composer’s emotions on the same scale (Cronbach’s α = 0.94). Dispositional empathy was measured with TEQ (Cronbach’s α = 0.87).

### Results

#### Manipulation check

Participants believed the composer to experience more positive emotions in the positive emotion (*M* = 5.50, *SD* = 1.73) than in negative emotion (*M* = 2.82, *SD* = 1.80, *F* (1, 210) = 124.12, *p* < .001) condition, regardless of participants’ dispositional empathy level (*p* = .97), suggesting that the manipulation of the composer’s emotions was successful and worked equally well for high- and low-empathy individuals.

#### Main analyses

**Fig. 5:**
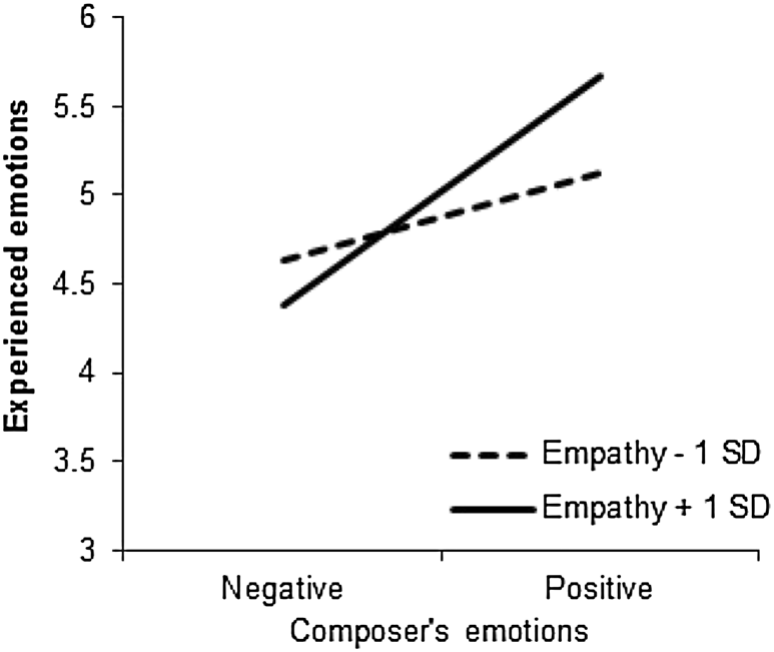
Participants’ experienced emotions (1 = negative, 7 = positive) as a function of their empathy level and emotions experienced by the composer, Study 2a

Means, standard deviations and zero-order correlations are shown in Table 1. To examine whether the composer’s emotions had a causal effect on participants’ emotional reactions to his music regardless of the music’s emotional valence and whether it was moderated by individual differences in empathy, we conducted a 2 (music valence: positive vs. negative) x 2 (composer’s emotions: positive vs. negative) x empathy (scale predictor, mean centered) ANOVA with participants’ experienced emotions as dependent variable. The model specified all main effects, all two-way interactions and the three-way interaction among the variables. The results revealed a significant main effect of the emotional valence of the music, *F* (1, 206) = 54.34, *p* < .001, η^2^_partial_ = 0.21. Participants experienced more positive emotions after listening to a positive valence piece (*M* = 5.56, *SD* = 1.19) than after listening to a negative valence piece (*M* = 4.33, *SD* = 1.43). The interaction between the emotional valence of the music and empathy was not significant, *F* (1, 206) = 2.02, *p* = .16. The main effect of the composer’s emotions reached significance, *F* (1, 206) = 27.70, *p* < .001, η^2^_partial_ = 0.11. Importantly, consistent with our prediction, it was qualified by a significant interaction with participants’ dispositional empathy level, *F* (1, 206) = 5.37, *p* = .021, η^2^_partial_ = 0.03. This interaction is presented on Fig. 5. It shows that the effect of the composer’s emotions on participants’ experienced emotions was stronger in participants with a high (+1 *SD*) empathy score (*M*_dif_ = 1.30, *p* < .001) than in participants with a low empathy score (−1 *SD, M*_dif_ = 0.50, *p* = .041). That is, after listening to a piece of music, high-empathy individuals were more likely to experience the emotions ostensibly experienced by the composer than low-empathy individuals, regardless of whether the emotions expressed by the music matched the composer’s emotions or not. These results provide support for our causal assumption, suggesting that the perception of an artist’s emotions is more likely to affect high- (compared to low-) empathy individuals’ emotional reactions to art. No other effects of interactions reached significance (all *p*s > 0.16).

#### Gender effects

As the composer was depicted as a male (wearing a male name), we explored whether the effect of the music valence and its interaction with empathy was stronger among males than females. We conducted a 2 (music valence: positive vs. negative) x 2 (composer’s emotions: positive vs. negative) x 2 (participants’ gender: male vs. female) x empathy (scale predictor, mean centered) ANOVA with participants’ experienced emotions as dependent variable. We found a significant interaction between gender and composer’s emotions, *F* (1, 197) = 6.21, *p* = .014, η^2^_partial_ = 0.02. Men were more likely to be affected by the artist’s emotions than women. The interaction between empathy and composer’s emotions was significant, *F* (1, 197) = 8.37, *p* = .004, η^2^_partial_ = 0.04, and did not depend on participants’ gender (the three-way interaction between empathy, artist’s emotions and participants’ gender was not significant, *F* (1, 197) = 2.09, *p* = .15). That is, high-empathy individuals were more likely to experience the emotions ostensibly experienced by the composer than low-empathy individuals, regardless of their gender. No other interactions reached significance (all *p*s > 0.12).

## Study 2b

In Study 2b, we replicated this effect in the domain of visual art: photography. Again, we manipulated the photographs’ emotional valence and the photographer’s emotions independently of each other and measured high- and low-empathy individuals’ experienced emotions after viewing the photographs. In addition, to rule out potential carry-over effects between participants’ responses to the artwork and empathy, we additionally counterbalanced the order in which empathy was measured (either before or after viewing the photographs).

### Method

We sampled 391 individuals from MTurk. 65 failed the manipulation check (the same as in Study 1b). The final sample consisted of 326 individuals (mean age 34.66, *SD* = 12.16, 51.8% males). They were randomly assigned to one out of eight experimental conditions in a 2 (photographs’ emotional valence: positive vs. negative) x 2 (photographer’s emotions: positive vs. negative) x 2 (item order: empathy measured at the start vs. at the end) between-subject design. To manipulate the photographer’s emotions, we used the same scenario as in Study 2a, describing a photographer Lucas M., who either lost his family or had a child born—the events he described as the happiest (vs. the worst) moment of his life. Participants were shown five either positive or negative emotional valence photographs Lucas M. ostensibly dedicated to his family (the same as in Study 1b) and rated their emotions after viewing each photograph (the same scale as in Study 1b was used, Cronbach’s α = 0.94). As a manipulation check, using the same scale, participants rated the emotions that Lucas M. could have experienced while taking these photographs. Like in previous studies, empathy was measured with TEQ (Cronbach’s α = 0.89).

### Results

#### Manipulation check

Participants rated the photographer’s emotions as more positive in the “happy photographer” (*M* = 4.75, *SD* = 1.90) than in the “sad photographer” (*M* = 2.47, *SD* = 1.76, *F* (1, 322) = 125.69, *p* < .001) condition. This effect did not depend on participants’ dispositional empathy level (*p* = .48), suggesting that the manipulation was successful and worked equally well for high- and low-empathy individuals.

#### Main analysis

**Fig. 6:**
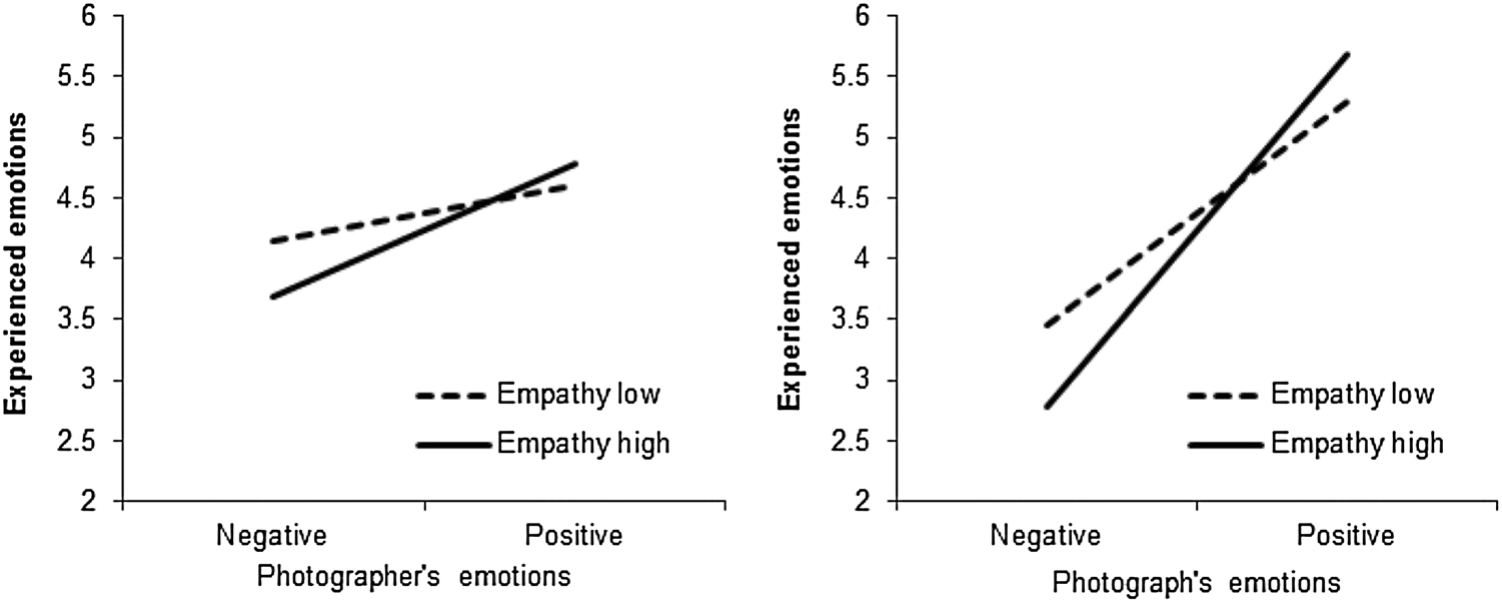
Effect of empathy and the emotions experienced by the photographer (*left panel*), and the emotional valence of the photographs (*right panel*) on participants’ experienced emotions (1 = negative, 7 = positive), Study 2b

Means, standard deviations and zero-order correlations are shown in Table 1. We conducted a 2 (photographs’ valence: positive vs. negative) x 2 (photographer’s emotions: positive vs. negative) x empathy (scale predictor, mean centered) x 2 (item order: empathy measured at the start vs. at the end) ANOVA with participants’ experienced emotions as dependent variable. The model specified all main effects, all two- and three-way interactions and the four-way interaction among the variables. The results showed a significant main effect of photographs’ emotional valence, *F* (1, 310) = 522.93, *p* < .001, η^2^_partial_ = 0.63. Participants experienced more positive emotions after viewing positive valence photographs (*M* = 5.52, *SD* = 0.95) than negative valence photographs (*M* = 3.13, *SD* = 1.12). This effect was qualified by a significant interaction with empathy, *F* (1, 310) = 25.96, *p* < .001, η^2^_partial_ = 0.08 (Fig. 6). The effect of photographs’ valence on participants’ experienced emotions was stronger in participants with a high (+1 *SD*) empathy score (*M*_dif_ = 2.91, *p* < .001) than in participants with a low empathy score (−1 *SD, M*_dif_ = 1.84, *p* < .001). The main effect of the photographer’s emotions reached significance as well, *F* (1, 310) = 56.31, *p* < .001, η^2^_partial_ = 0.15. Importantly, it was also qualified by a significant interaction with empathy, *F* (1, 310) = 9.34, *p* = .002, η^2^_partial_ = 0.03 (Fig. 6). The effect of the photographer’s emotions on participants’ experienced emotions was stronger in participants with a high (+1 *SD*) empathy score (*M*_dif_ = 1.10, *p* < .001) than in participants with a low empathy score (−1 *SD, M*_dif_ = 0.46, *p* < .01). As indicated by a non-significant interaction among empathy, the emotional valence of the photographs and the photographer’s emotions (*p* = .58), empathy’s effect on the degree to which participants’ emotions were influenced by the photographer’s emotions and the emotions expressed on the photographs were independent of each other. Also, these effects did not depend on whether empathy was measured at the beginning or at the end of the study, suggesting that potential carry over effects did not affect our findings (all *p*s > 0.23).

#### Gender effects

As the photographer was depicted as a male (wearing a male name), we conducted a 2 (photographs’ valence: positive vs. negative) x 2 (photographer’s emotions: positive vs. negative) x 2 (participants’ gender: male vs. female) x empathy (scale predictor, mean centered) ANOVA with participants’ experienced emotions as dependent variable. These analyses revealed a significant interaction between empathy and photographs’ valence, *F* (1, 308) = 18.83, *p* < .001, η^2^_partial_ = 0.06, as well as between empathy and photographer’s emotions, *F* (1, 308) = 6.23, *p* = .013, η^2^_partial_ = 0.02. Gender had no main effect, neither did it interact with any other factor in these analyses (all *p*s > 0.13).

Overall, high-empathy individuals were more likely to experience the emotions both expressed in the photographs and ostensibly experienced by the photographer than low-empathy individuals. These effects were independent of each other. These results support our causal model in which the perception of an artist’s emotions represents a causal factor leading to a stronger emotional reaction to his/her artworks among high- (vs. low-) empathy individuals.

## Discussion

The goal of Studies 2a and 2b was to address the causality of the association between high-empathy individuals’ perception of the artist’s emotions and their own emotional reactions to the artwork, established in Studies 1a and 1b. We experimentally manipulated the artist’s emotions and found that after contemplating an artwork, high-empathy individuals were more likely to experience the emotions ostensibly experienced by the artist than low-empathy individuals. This effect emerged with respect to both music (Study 2a) and photographs (Study 2b) and did not depend on what emotions were expressed by the artwork.

At the same time, at least with respect to the photographs (Study 2b), high-empathy individuals were more likely to catch the emotions expressed by the artwork than low-empathy individuals (Study 2b), regardless of the emotions ostensibly experienced by the artist. This interaction did not emerge with respect to music (Study 2a). We speculate that the effect of the artworks’ emotional valence was more pervasive in the photographs’ study, as participants were exposed to five stimuli, whereas in the music study, they only listened to one piece of music. Besides this difference, both studies provided evidence for the causal links between high-empathy individuals’ perception of the artist’s emotions and their emotional reactions to his/her artworks.

## Study 3

In studies 1a through 2b, we manipulated the emotional valence of the artwork via selecting the pieces that were identified as expressing positive or negative emotions in previous studies. While this procedure allows to study people’s reactions to the emotions expressed in the artwork, it does not allow to explore people’s reactions to the emotions actually experienced by the artist during the creation process. Are high-empathy individuals better at identifying the emotions experienced by the artist based on a brief exposure to their work than their low-empathy counterparts? While contemplating an artwork, will highly empathetic individuals be more likely to take on the emotions the artist was experiencing while creating it? Study 3 was designed to answer these questions.

Also, compared to previous studies, in Study 3, we used another type of art—poetry. Based mainly on convenience considerations, we chose a major form of Japanese poetry, known as haiku. Haiku is a very short poetry form, usually written in three unrhymed lines. As we needed to have experimental control over the emotions experienced during the creation process, we recruited individuals to write haikus as part of a psychological research study. Before the writing task, we experimentally induced positive vs. negative emotional states using the autobiographical recall procedure (Forgas 1999). The poems were then shown to another sample of participants who rated their own and the “artists” emotions. We examined whether high- (vs. low-) empathy individuals will be better able to identify and take on the emotions experienced by the “artists”.

Finally, to examine whether our findings apply to cognitive and/or affective facets of empathy, additionally to the Toronto Empathy Scale used in Studies 1a through 2b, we included Empathetic Concern (affective empathy) and Perspective Taking (cognitive empathy) subscales of the Interpersonal Reactivity Index (Davis 1983).

### Method

#### Materials’ generation

To allow a certain degree of generalizability, we aimed to use ten poems in each condition (20 different poems altogether). As we did not know how many participants would provide usable material, we decided to recruit app. twice as many “artists” as we actually needed, ending up with 51 individuals recruited on MTurk. The study was described as consisting of two unrelated parts: part one was about memory and part two was about artistic experience and included a task to “write a brief literary text”. In the first part, participants were asked to remember a specific social event that has occurred in their life that has made them either very happy (positive emotions condition) or very sad (negative emotions condition) (Forgas 1999). They were asked to describe this event in at least six sentences and afterwards responded to a couple of filler questions about the event and, as a manipulation check, rated their current mood using a seven-point scale [‘happy’ and ‘sad (reverse-coded)’, Cronbach’s α = 0.87]. Participants in the happy condition reported more positive emotions (*M* = 6.09, *SD* = 1.02) than participants in the sad condition (*M* = 3.08, *SD* = 1.68; *t* (37.026) = 7.62, *p* < .001).

Afterwards, participants were introduced to a Japanese poetry form—haiku. They read that “a haiku uses just a few words to capture a moment and create a picture in the reader’s mind. It is like a tiny window into a scene.” Participants were told that haikus are typically written in three lines and were asked to compose one haiku. As our participants were not professional poets, we released further requirements (pertaining to the number of syllables). We obtained 48 usable haikus overall.^3^ For the main study, we used ten positive and ten negative emotionality haikus, written by the authors who scored the lowest (in the negative emotions condition) versus the highest (in the positive emotions condition) on the mood manipulation check question. An example of a sad haiku is “The snow falls softly. The chill has crept inside me. There is no comfort.” An example of a happy haiku is “See small rabbits. Hopping around in the grass. Nibbling hungrily”.

#### Main study

We recruited 404 (mean age 36.91, *SD* = 12.39, 43% females) individuals from MTurk to participate in a study on poetry evaluation. MTurk workers who participated as “artists” in materials’ generation phase were not eligible for this study. Participants read ten haikus of either positive or negative emotional valence. Within each condition, the haikus were presented in a random order. After each haiku, using a scale ranging from 1 = ‘not at all’ to 7 = ‘a lot’, participants indicated how they thought the author was feeling while writing it [‘happy’ and ‘sad (reverse-coded)’, Cronbach’s α = 0.85] and, using the same scale, stated their current emotions (Cronbach’s α = 0.94). The responses were averaged across the haikus. At the end, participants completed the TEQ scale (1 = ‘rarely’, 4 = ‘always’; Cronbach’s α = 0.89) and the Empathetic Concern (EC) and Perspective Taking (PT) (1 = ‘does not describe me well’, 5 = ‘describes me very well’; Cronbach’s α = 0.89 and 0.86, respectively) subscales of the Interpersonal Reactivity Index (Davis 1983).^4^

### Results

Means, standard deviations and zero-order correlations among measured variables are shown in Table 2. TEQ was very strongly related to EC (*r* = .89, *p* < .001) and PT (*r* = .64, *p* < .001), the latter were also related to each other (*r* = .57, *p* < .001). Higher empathy scores on all three scales were related to more positive perceived emotions in general (0.19 ≤ *r* ≤ .23, *p* < .001); higher levels of TEQ and EC were associated with more positive experienced emotions (*r* = .11, *p* < .05 and *r* = .14, *p* < .01, respectively).

**Table 2:** Means, standard deviations and zero-order correlations among the variables, Study 3

		*M*	*SD*	1	2	3	4	5
1	TEQ	0.00	0.62	–	–	–	–	–
2	EC	3.76	0.87	0.89***	–	–	–	–
3	PT	3.70	0.76	0.64***	0.57***	–	–	–
4	Experienced emotions	4.93	1.09	0.23***	0.19***	0.22***	–	–
5	Perceived emotions	4.27	0.88	0.11*	0.14**	0.07	0.50***	–
6	Poems’ valence (manip. factor)	0.51	0.50	0.05	0.08	0.02	0.31***	0.81***

***p < .001, ** *p* < .01, * *p* < .05. *TEQ* Toronto empathy questionnaire, *EC* empathetic concern, *PT* perspective taking

We conducted a 2 (poems’ valence: positive vs. negative) x TEQ (scale predictor, mean centered) MANOVA with experienced emotions and perceived emotions as dependent variables. The model included the main effects of poems’ valence and TEQ and the two-way interaction between them. The omnibus test revealed significant main effects of poems’ valence (Pillai’s Trace *F* (2, 399) = 410.35, *p* < .001, η^2^_partial_ = 0.67), TEQ (Pillai’s Trace *F* (2, 399) = 11.49, *p* < .001, η^2^_partial_ = 0.05) and a significant interaction between them (Pillai’s Trace *F* (2, 399) = 10.24, *p* < .001, η^2^_partial_ = 0.05).

#### Perceived emotions

**Fig. 7:**
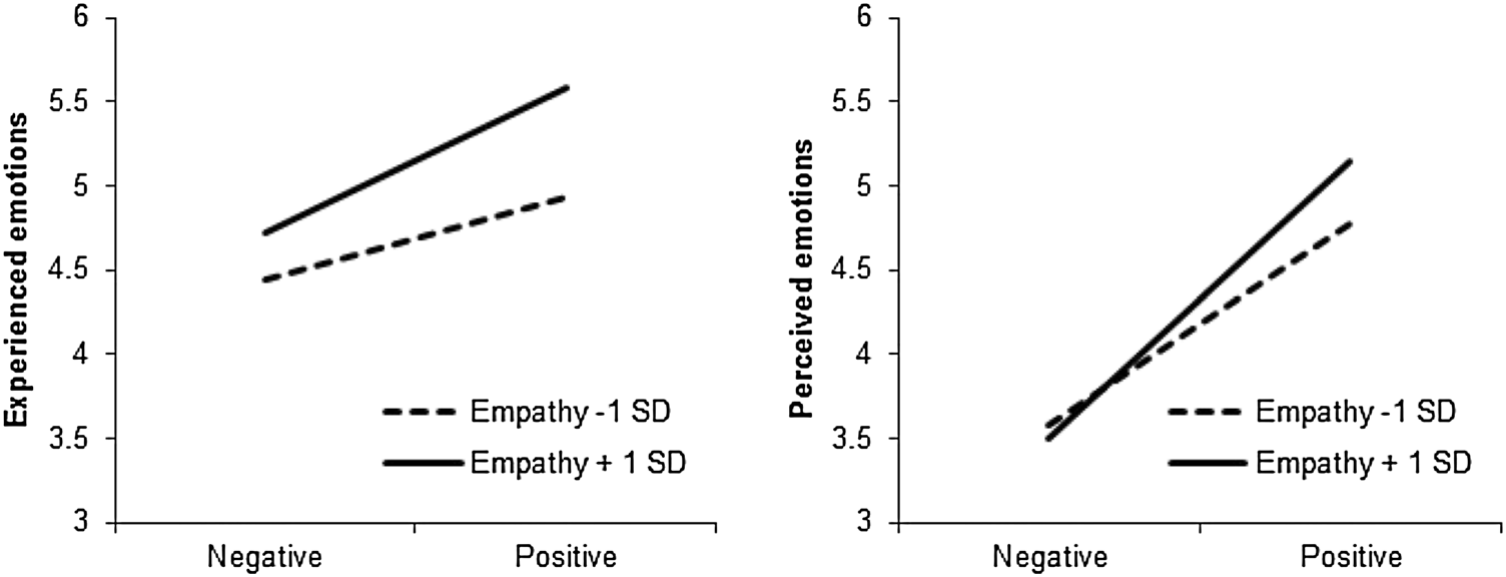
Participants’ experienced and perceived emotions (1 = negative, 7 = positive) as a function of their empathy level (TEQ) and the emotional valence of the poems, Study 3

Participants believed that the “artists” experienced more positive emotions after reading positive valence (*M* = 4.96, *SD* = 0.49) than negative valence haikus (*M* = 3.54, *SD* = 0.54), *F* (1, 400) = 794.94, *p* < .001, η^2^_partial_ = 0.67. This effect was stronger for high- (*M*_dif_ = 1.64, *p* < .001) than for low- (*M*_dif_ = 1.19, *p* < .001) empathy individuals, *F* (1, 400) = 20.53, *p* < .001, η^2^_partial_ = 0.05 (Fig. 7).

The analyses using EC and PT (instead of TEQ) showed nearly identical results. The effect of poems’ valence on perceived emotions was stronger for individuals scoring 1 *SD* above average (*M*_dif_ = 1.58, *p* < .001) than for individuals scoring 1 *SD* below average on EC (*M*_dif_ = 1.24, *p* < .001), *F* (1, 400) = 10.61, *p* = .001, η^2^_partial_ = 0.03. The interaction between poems’ valence and PT was significant as well, *F* (1, 400) = 10.88, *p* = .001, η^2^_partial_ = 0.03. The effect of poems’ valence was stronger among individuals with higher (+1 *SD*) (*M*_dif_ = 1.60, *p* < .001) than lower (−1 *SD*) (*M*_dif_ = 0.05, *p* = .91) PT scores.

#### Experienced emotions

Participants who read positive emotional valence poems reported more positive emotions (*M* = 5.27, *SD* = 0.98) than participants who read negative emotional valence (*M* = 4.57, *SD* = 1.09) poems. As suggested by a marginally significant interaction between TEQ and poems’ valence, *F* (1, 400) = 3.40, *p* = .066, η^2^_partial_ = 0.01, this effect was stronger for high- (*M*_dif_ = 0.87, *p* < .001) than for low-empathy (*M*_dif_ = 0.49, *p* < .01) individuals (Fig. 7).

The same results were obtained with EC (instead of TEQ). The interaction between EC and poems’ valence was marginally significant, *F* (1, 400) = 3.40, *p* = .066, η^2^_partial_ = 0.01, and the effect of the stimuli valence was stronger for high- (*M*_dif_ = 0.86, *p* < .001) than for low-EC (*M*_dif_ = 0.48, *p* = .001) individuals. Yet, in contrast to EC and TEQ, the interaction between PT and poems’ valence did not reach significance, *F* (1, 400) = 0.35, *p* = .55.

#### Moderated mediation analysis

**Fig. 8:**
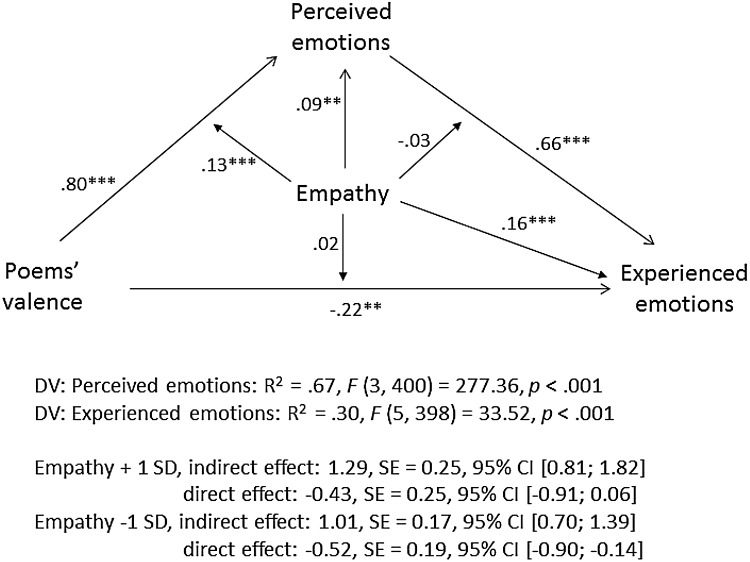
Moderated mediation analysis with TEQ as a measure of empathy, Study 3. Note. The diagram paths present the standardized regression coefficients, the estimation of the indirect effects is based on unstandardized coefficients (Hayes 2016)

**Fig. 9:**
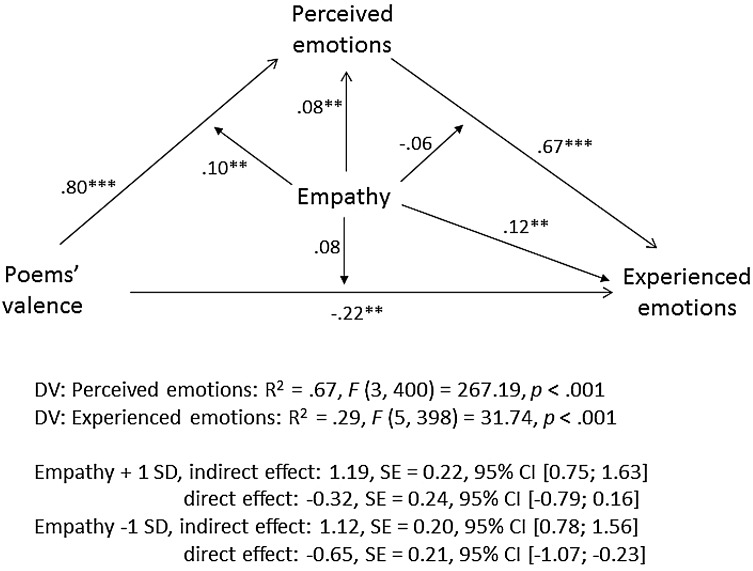
Moderated mediation analysis with EC as a measure of empathy, Study 3. Note. The diagram paths present the standardized regression coefficients, the estimation of the indirect effects is based on unstandardized coefficients (Hayes)

Using exactly the same procedure as in Study 1a, we conducted a moderated mediation analysis with poems’ emotional valence as independent variable, experienced emotions as dependent variable, perceived emotions as a mediator, and TEQ as a moderator. The model’s coefficients are shown in Fig. 8. Among high-empathy individuals (+1 *SD*), the conditional indirect effect of poems’ valence on experienced emotions via perceived emotions was *b* = 1.29, *SE* = 0.25, 95% CI (0.81; 1.82); whereas among low-empathy individuals (−1 *SD*), it was considerably smaller, *b* = 1.01, SE = 0.17, 95% CI (0.70; 1.39). As indicated by a significant interaction between poems’ emotional valence and empathy (*ß* = 0.13, *p* < .001), high-empathy individuals were more likely to correctly identify the emotions experienced by the “artists” than low-empathy individuals and, consequently, were more likely to experience these emotions themselves. Nearly identical results were obtained when EC was used instead of TEQ (s. Fig. 9). We didn’t conduct the moderated mediation analyses with PT, as there was no interaction between PT and poems’ valence on experienced emotions.

## Discussion

Study 3 extended the results of Studies 1a–2b in a number of ways. First, it showed that empathy facilitates accurate identification and experience of emotions not only in music (Studies 1a and 2a) and photographs (Studies 1b and 2b), but also in literary texts. Second, it demonstrated that empathy is not only related to a higher sensitivity to emotions expressed in artworks but also to emotions actually experienced by the artist during the creation process. Third, by including the measures of empathetic concern and perspective taking, this study helped to disentangle the role of affective versus cognitive empathy in these processes. Our results suggest that only affective empathy was associated with a stronger tendency to take on the emotions experienced by the artist, whereas both affective and cognitive facets of empathy were positively related to empathetic accuracy in the context of art. This finding is consistent with prior research showing that the affective facet of empathy predicts empathetic accuracy in the context of emotion recognition from videos (Zaki et al. 2008).

## General discussion

Empathy has traditionally been linked to individuals’ ability to understand and take on other people’s emotions. High-empathy individuals are more likely to correctly identify others’ emotions from their facial expressions and to feel others’ affect as their own compared to low-empathy individuals (Baron-Cohen et al. 2001; Dimberg et al. 2011; Svetieva and Frank 2015). The present research suggests that these effects are not restricted to social targets, such as faces, but extend to products of the human mind, such as objects of art. We proposed that in the same way as high dispositional empathy directs individuals’ attention to emotional cues on others’ faces (Hofelich and Preston 2012), it can help individuals perceive and react to emotional cues contained in artworks. Across five experiments, we have shown that high-empathy individuals are more likely to use the emotional information contained in the artwork to make inferences about the emotions experienced by the artist, compared to their low-empathy counterparts. Consequently, high-empathy individuals are more likely to get “contaminated” by the emotions expressed in the artwork, that is, more likely to become subject to emotional contagion through art. These results were obtained with respect to three different art forms—music, photography and poetry. The underlying mechanism—inferences about the artist’s emotions—has been demonstrated using two different methods: a measurement-of-mediation and experimental-causal-chain mediation designs (Spencer et al. 2005). In Studies 1a and 1b, we manipulated the emotions expressed in music (Study 1a) and photographs (Study 1b) and using a measurement-of-mediation approach, showed that high-empathy individuals were more likely to infer the artist’s emotions based on the emotional valence of the artwork and the perception of the artist’s emotions affected their own emotional reactions to the artworks. In Studies 2a and 2b, we complemented these results using an experimental-causal-chain mediation design: we experimentally manipulated the emotions experienced by the artist and showed that they are more likely to affect high- rather than low-empathy individuals’ emotional reactions to art. Finally, in Study 3 we showed that empathy does not only contribute to an accurate identification of the emotions expressed in artworks, but also facilitates the identification of emotions experienced by the artist while creating an artwork. We manipulated participants’ emotions and asked them to write brief literary texts that were then shown to another sample of participants. Overall, this second group of participants could identify the emotions experienced by the “artists” at a better than chance level and participants with high scores on cognitive or affective empathy did a better job than their less empathetic counterparts. In addition, individuals with a high level of affective (but not cognitive) empathy were slightly more likely to experience the emotions experienced by the “artists”, although this effect was significant at just a 6.6% level. It is also important to note that the effect of poems’ valence and its interaction with empathy on experienced emotions in this study was substantially smaller, compared to Studies 1a–2b. These smaller effect sizes are not surprising though, as our “artists” were not asked to express their currently experienced emotions in their poems. Prior research has shown that targets’ expressivity might represent a crucial component driving the empathy effect. Specifically, Zaki et al. (2008) has shown that high-empathy individuals’ increased ability to accurately identify others’ emotions based on video-recorded samples of behavior is restricted to particularly expressive targets. It might be interesting to explore in future studies whether the effect of trait empathy on empathetic accuracy and emotional contagion through art depends on the expressivity of the artwork as well.

On a related note, emotions that artists express in their work might not necessarily correspond to the emotions they experience while working on an art object. For example, a musician experiencing a personal drama might be commissioned to compose a cheerful piece for a wedding or another celebration. Will high-empathy individuals outperform their low-empathy counterparts in identifying emotions experienced by the artist while creating (or performing) an artwork, even if these experienced emotions don’t correspond to the expressed ones? It might be worth examining whether empathy does not only predict individuals’ ability to recognize emotions expressed in art but also to correctly identify cases when expressed emotions are not experienced by the artist.

The moderated mediation analyses shown here (Figs. 2, 4, 8, 9) deserve particular attention. These analyses showed that participants’ perceived emotions mediated the effect of stimuli emotional valence on their experienced emotions and that this mediation effect was stronger among high- than low-empathy individuals. Indeed, among high-empathy individuals, perceived emotions fully mediated the effect of artworks’ valence on experienced emotions, yielding a non-significant direct effect. Yet, in Studies 1b and 3 (but not in Study 1a), among low-empathy individuals, the direct effect even turned negative, pointing at a potential suppression effect (MacKinnon et al. 2000). When holding their perception of the artist’s emotions constant, low-empathy individuals seemed to react negatively to positive valence stimuli. In other words, among low-empathy individuals, those who were in the positive valence condition but reported the same “perceived” emotions as those in the negative valence condition, tended to experience negative rather than positive emotions themselves. We tend to interpret it in the following way: low-empathy individuals were probably just more likely to misperceive expressed positive emotions as negative (and another way around), which affected their own experienced emotions. However, given that this pattern of suppression was not consistently observed across all studies, more research is necessary to better understand its origin.

While this research has highlighted the role of high-empathy individuals’ beliefs about artists’ emotions as a mechanism underling their emotional reactions to art objects, there might be other mechanisms at work as well. For example, in the case of performing arts, such as music or dance, individuals can use the emotional information contained in the artwork to infer the performer’s (rather than the creator’s) emotions or both. In fact, prior studies have shown high-empathy individuals to be sensitive to the emotions expressed by performing musicians (Engel and Keller 2011). An interesting question for future research on aesthetic appreciation of art might be to pit the creator’s versus the performer’s emotions against each other to determine which one plays a primary role in driving observers’ emotional reactions to art.

The present studies are not without limitations, with online samples and self-report measures of emotions being the most important ones. Hence, we encourage future research to replicate these results using different samples and behavioral or physiological measures of emotions. In addition, while the present research focused on emotional valence in general, it might be interesting to explore whether trait empathy can facilitate contagion of discrete emotions, such as anger or fear. Finally, even though we used the term “art” to refer to the pieces of music, photographs and poems in our studies, only the former (Studies 1a and 2a) fall under the category of art, in a strict sense of the word. The photographs used in Studies 1b and 2b were not taken from an art gallery or an art album and the haikus that our participants created in a 10-min study would not satisfy all the criteria that professional haikus must fulfil and would probably not be recognized as art by literary experts. Yet, drawing from psychological research on art, a creation’s quality or expert judgement are not crucial for this piece of work to be perceived as art by others (Preissler and Bloom 2008). On the other hand, although we used the term “artwork” to describe the subject of our research, we believe that the empathy effect we demonstrated is not restricted to objects labeled “artworks” but can exist with respect to probably just any human-made artifacts, as long as such artifacts express their creators’ emotions. We hope future studies would explore this possibility empirically.

Demonstrating the importance of empathy in emotional reactions to non-social targets, the present results are of theoretical importance for a number of psychological sub-disciplines, including the study of emotions, personality and the psychology of arts and aesthetics. At the same time, our results might be of methodological importance. Specifically, psychological research traditionally uses music and pictures (including photographs from the International Affective Picture System used here) as a method of mood induction. Our results pointed at substantial inter-individual variability in the effectiveness of these methods: high-empathy individuals were more likely to subsume to the manipulation than low-empathy individuals. These findings suggest that it might be wise to take individual differences in empathy into consideration in studies using these mood manipulation techniques.

To conclude, the largest majority of empirical work on empathy attempted to understand its role in interpersonal relationships. In contrast, in the present research, continuing the tradition set at the dawn of research on empathy over a century ago (Lipps 1903), we showed that the role of trait empathy extends much beyond interpersonal relations and gives rise to emotional contagion through non-social targets.

## Footnotes

1 Filler items included participants’ favourite music genre, whether they had any musical training and similar questions. For exploratory reasons, we measured two further emotions (angry and fearful) and trait emotional contagion (only in Studies 1a and 2a). All the materials (incl. questionnaires) and the data of all studies reported here can be accessed online (removed for review, 2017).

2 We preferred the analysis of variance to regression out of convenience: it allows examining participants’ experienced and perceived emotions at once and is consistent with the method used in further studies in this paper (that included two manipulated factors and the analysis of variance is even more convenient in such cases). However, note that regression provides identical results.

3 Three participants produced unusable poems (e.g., Yep. Nope. Great“).

4 Due to the specifics of the materials used, we expected the effect size to be smaller than in studies 1a to 2b and collected 203 participants. Unfortunately, we noticed a programming error then: the three items that TEQ and EC have in common were only included in the latter and measured on a 5-point scale like other EC items, while TEQ items used a 4-point scale. To compute the TEQ score, we standardized all TEQ items. To be sure about our results, we corrected the programming error and collected further 201 observations. We then combined both samples. Our results do not depend on how TEQ was computed (the way it was computed had neither main effect on any dependent variable, nor did it interact with any other variable in the analyses, all *p*s > 0.40).
